# How to Start WALANT Practice in South Africa: “Service With a Smile if You Are Willing to Wait Awhile.”

**DOI:** 10.1016/j.jhsg.2022.05.010

**Published:** 2022-06-22

**Authors:** Odette Koch

**Affiliations:** ∗ Department of Orthopaedic Surgery, 1 Military Hospital, South African Military Health Service, Pretoria, South Africa

**Keywords:** Cost effectiveness, Pandemic, Patient experience, WALANT, Wide-awake hand surgery

## Abstract

Wide-awake local anesthesia no tourniquet (WALANT) hand surgery at 1 Military Hospital in South Africa resulted in a positive patient experience and beneficial financial outcomes in an economically constrained environment. Using the WALANT technique also effectively reduced the waiting time for elective surgery, which is considered to be synonymous with improvement in the public health sector in South Africa. Elderly patients and those medically unfit for general anesthesia successfully underwent WALANT surgery and returned to daily activities. Additionally, the South African WALANT surgery initiative established a successful outreach program with Operation Healing Hands. Continuation of WALANT procedures during the pandemic in a “do not touch” environment had a low probability for disease transmission and was advantageous in reducing the elective surgery workload. In the beginning, WALANT’s initiation at the institution was brutal. Eventually, the critics shifted to value the inexpensive, effective service delivery of the WALANT method. Flexibility, open mindedness, and resilience are necessary to implement wide-awake surgery initiatives successfully.

The South African Hand Society for Surgery of the Hand (SASSH) has a rich history. It was established on July 11, 1969.^1^ SASSH is also proud to be among the earliest members of the International Federation of Societies for Surgery of the Hand. Over the years, many distinguished hand surgeons have visited our country. Among these, in the early years, were Guy Pulvertaft, Daniel Riordan, and Willy White. In more recent years, world-renowned hand surgeons honored our society with visits to our congresses. South Africa is in the same time zone as most of Europe. Owing to the time zone differences and long traveling times between Asia, the Americas, and Canada, visits from fellow surgeons from these countries are few and far between.

Both Jin Bo Tang from China and Donald Lalonde from Canada traveled halfway across the globe to our congresses in recent years. The field of wide-awake, local anesthesia, no tourniquet (WALANT) surgery used to be uncharted territory in South Africa. When Jin Bo Tang introduced SASSH to the concept of WALANT, we frowned. Jin Bo Tang presented their experience from 2 units in China of more than 12,000 cases.^2^ Evidence showing epinephrine’s safety for use in hands, the improved work efficiency, high patient satisfaction, and improved patient outcomes still did not convince the South African surgeons to start using WALANT.^3^, ^4^, ^5^, ^6^, ^7^, ^8^ Things changed when Donald Lalonde visited South Africa in 2019 and had a similar message to that of Jin Bo Tang. His presentation and outcome videos made some of the surgeons in the audience reconsider WALANT surgery.

The inception of WALANT procedures at a military hospital in South Africa has had a fruitful and financially beneficial result. The South African Military Health Service (SAMHS) is the larger South African National Defence Force (SANDF) medical division. The SANDF comprises the South African Air Force, South African Army, South African Navy, and SAMHS. All the members of the SANDF and their dependents receive medical attention at a military facility, either a local sick bay or at 1 of the 3 military hospitals. The most important of the 3 facilities is 1 Military Hospital (1MH).

The orthopedic department at 1MH has a large catchment area. It treats orthopedic emergency cases and all elective surgical needs of the members and dependents of Gauteng and the surrounding provinces, including Limpopo, North West, Mpumalanga, and Kwa Zulu Natal, as well as some oncology and arthroplasty needs from the Northern Cape and Free State Province. The Orthopaedic Outpatient Department (OOPD) attended to more than 17,000 patients per year before the COVID-19 pandemic. Like most government hospitals in South Africa, the waiting lists for elective surgery far outweigh the available theater time.

## The Setting of the WALANT Practice at 1MH

The concept of performing surgery in the outpatient department is a paradigm shift. After Don Lalonde’s visit in 2019, the idea of moving small hand cases out of the theater complex and performing surgery in the clinic was proposed to the institutions’ General Officer Commanding. The Department of Defence Intelligence and the 1MH Research and Ethics Committee also had to approve implementing such a notable change. Colleagues and role players resisted the move of hand cases to the OOPD. We had to jump through a considerable number of hoops to make it happen. The military is, furthermore, a cost-conscious environment. The positive economic effect as experienced in a military set-up in the United States swayed the decision-makers to authorize the implementation of WALANT, provided that we emphasized patient safety.^9^

**Figure 1:**
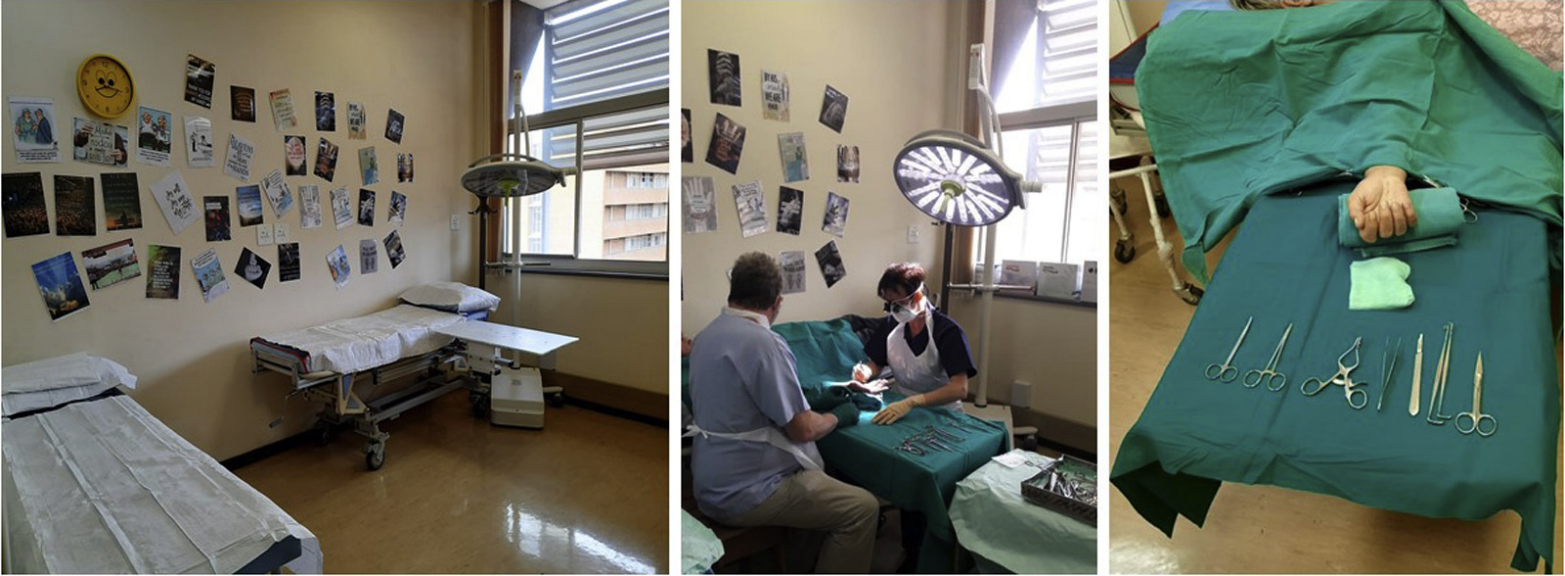
A room in the OOPD was furnished with the essential requirements. The Central Sterile Services Department supplies the OOPD with the required handsets for a list.

A room in the OOPD was furnished with the essential requirements.^10^ The Central Sterile Services Department supplies the OOPD with the required handsets for a wide-awake list. A routine WALANT list is limited to 8 cases per day, as the hospital has 12 handsets, and some sets should be available for the main operating theater complex. There is no autoclave available in the OOPD (Fig. 1).

Initially, the patients were skeptical about the concept of wide-awake surgery in the clinic. We created a patient information leaflet to inform the patients about the dos and don’ts of WALANT. The patients were especially skeptical about the ability to eat before the scheduled procedure. We also included information on driving after the surgery, wound care, follow-ups, and other shared concerns.

## Results of the Successful Implementation of WALANT at 1MH

### Positive patient experience

With the approval of the 1MH research and ethics committee, we performed a study regarding the subjective patient experience with the WALANT method.^11^ The purpose of the study was to quantify the 100 patients’ experiences, including pain, as reported on the visual analog scale, for the WALANT injection (1.5; standard deviation, ±1.6) and the operation (0.2; standard deviation, ±0.7). Patients who received either a carpal tunnel release or a trigger finger release in the clinic were included. The patients in the study population chose to receive surgery by the WALANT method in the clinic rather by than the conventional method under general anesthesia in the theater. The patients’ feedback was extremely positive in the study. The patients advertised the wide-awake procedure to other patients, with the result that patients now request WALANT surgery for almost any pathology (Videos 1 and 2, available on the Journal’s website at www.jhsgo.org ).

### Reduced waiting time for elective surgery

Moving the small hand cases from the waiting lists in the general theater complex to the clinic positively impacted the exceptionally long waiting time for elective surgery that is synonymous with the government health sector in South Africa.^12^ This change reduced the waiting time for upper-limb cases to be performed under general anesthesia at 1MH from 3 to 4 months to 6 weeks.

African time is often defined as the unique tendency in parts of Africa toward a more relaxed attitude to time. The waiting lists for elective surgery in state practice are often based on “African time.” The waiting time in state practice, based on the experience of Groote Schuur Hospital, can vary from 0 to 1,880 days.^13^ The slogan “service with a smile if you are willing to wait a while” alludes to this calm and relaxed attitude.

### Patients who are at high risk for anesthesia and geriatric patients received care

Patients previously disqualified for any procedure received their care using WALANT. With minor hand surgery, we helped patients who had struggled for years, including patients with severe tracheal stenosis, poor cardiac ejection fraction, and lung cancer on home oxygen, to name but a few (Video 3, available on the Journal’s website at www.jhsgo.org). Before WALANT, the work-ups for these high-risk, medically unfit patients often resulted in excessive tests, like a cardiac ultrasound and an extended blood work-up, adding to the cost but failing to assist with a minor pathology, like a painful trigger thumb. We helped a 90-year-old patient with single tendon transfer surgery after an extensor pollicis longus rupture of a conservatively treated distal radius fracture. She could not crochet anymore before the surgery. An extensor indicis proprius to extensor pollicis longus transfer using WALANT allowed her to return to her hobby and improved her quality of life (Video 4, available on the Journal’s website at www.jhsgo.org ).

### Cost-effectiveness

The 1MH WALANT surgery initiative saves the institution R18892–R27962 ($1,220–$1,807) per procedure, according to the standard operating procedure for procedures performed in the general theater complex (Table). The institution’s standard operating procedure before WALANT required all procedures to be performed in the main operating theater, mostly under general anesthesia. The patients were admitted the day before the surgery, and baseline, preoperative tests for anesthesia were performed for all patients exceeding 40 years of age, regardless of the type of surgery. These small hand cases were performed at the end of the operation list, adding to patient morbidity and excessive periods of fasting in the elderly population.

**Table:** Cost Outline of Theater Standard Operating Procedure Work-Up at 1MH (March 2021)

Tests	Costs
Average hospitalization cost (includes 45 min theater time, excludes overnight)	R13550 ($872; range, R10100–R17000; $650–$1,095)
Full blood count and urea & electrolytes	R615 ($39)
Electrocardiogram	R122 ($8)
Chest X-ray	R642 ($41)
Lung function test	R407 ($26)
Extended biochemistry (selected cases)	R673 ($43)
Heart sonar (selected cases)	R1700 ($109)
Average anesthetist fee	R3894 ($250; range, R2993–R5351; $193–$383)

This sizable cost-benefit is undisputed and is similar to those reported in studies by Kurtzman et al,^8^ Rhee et al,^9^ and van Demark et al.^14^ The SANDF funding model is unique, and cannot be extrapolated to the rest of the state practices or to private health care across South Africa. Nonetheless, in a low income country and an economically strained health care system, the notable cost saving provides a tangible financial benefit.

### Outreach

Stumbling blocks, like access to surgical care, transport constraints, a lack of facilities in state practice, and a lack of adequate theater time for elective procedures, add to the burden of surgical diseases. These barriers in health care are uniform in Africa.^12^, ^13^, ^15^ The low cost of the WALANT injection (R202.34; $13) and limited requirements for field sterility and basic instruments make minor hand surgery more accessible with the help of an established outreach system. Operation Healing Hands is a charity initiative organized by doctors and ancillary medical professionals who aim to close the socioeconomic gap and provide surgery to patients in need.^16^ Operation Healing Hands focuses on addressing the health care services problem in South Africa. The organization provides a platform for medical professionals to help relieve the burden on government hospitals and improve the quality of life for as many patients as possible. In collaboration with the Triomf Clinic in the west of Pretoria, Operation Healing Hands provides WALANT surgery regularly to patients in need. The patients apply to Operation Healing Hands or are referred to the Triomf Clinic by a doctor from one of the neighboring state facilities. The procedures are performed in an office setting, and the patients are operated on and followed up pro deo by the responsible surgeons. Various other WALANT outreach projects are developing in South Africa, Kwa Zulu Natal’s northern region, and some neighboring countries.

### Paradigm shift

The initiation of WALANT at 1MH was challenging, and the amount of resistance from colleagues and important decision-makers was tremendous. Coworkers objected to the unthinkable idea of operating on patients who were awake, in a clinical environment with epinephrine on an extremity. Specialists waited for the infection complications to arise. Having a local champion is often needed to be the catalyst in this type of initiative. Eventually, WALANT at 1MH caused a paradigm shift in low-cost, effective service delivery with the patient’s best interest at heart. The mind shift of having a 1MH WALANT OOPD room resulted in 300 cases being operated, with a high patient satisfaction rate and an acceptable infection rate.^11^ The office setting is safe for minor procedures of the hand.^14^^,^^17^^,^^18^

### COVID-19 pandemic

**Figure 2:**
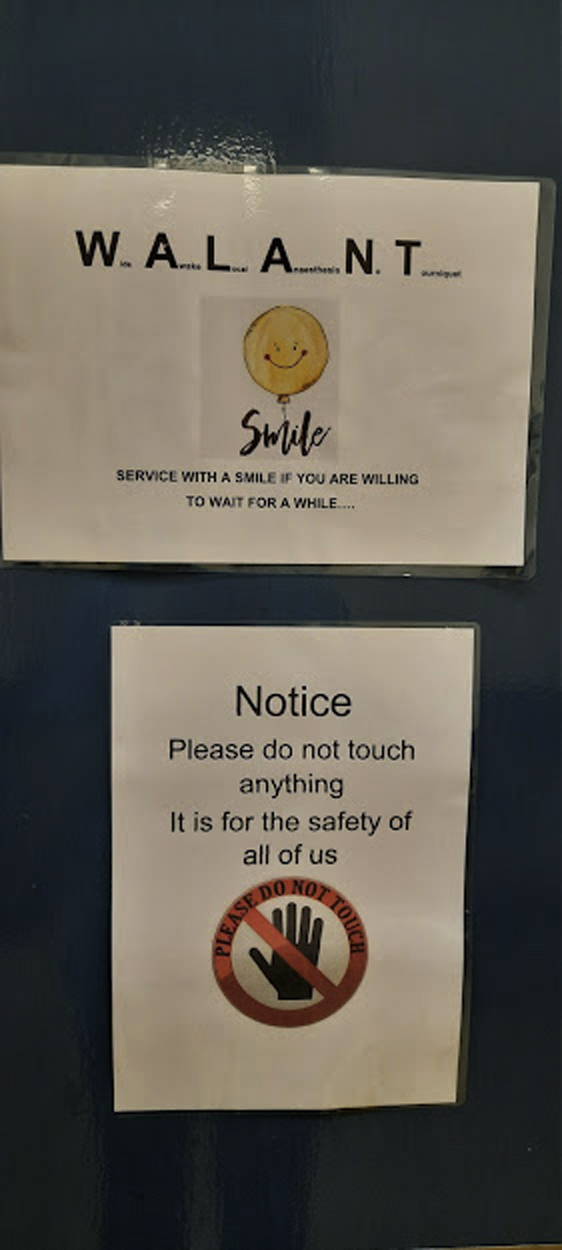
The WALANT room became a “do not touch” space after the national government lifted the initial lockdown level 5 in South Africa in May 2020. The patients would sanitize their hands upon entering.

The SANDF had various nationwide COVID-19 deployment obligations throughout the pandemic, resulting in a skeleton staff at the institution and the suspension of elective surgery until August 2020. We changed the WALANT room to a “do not touch” space after the national government lifted the initial lockdown level 5 in South Africa in May 2020. The patients would sanitize their hands upon entering. We used disposable linens, fresh airflow, surface sanitization, and adequate social distancing in the waiting area, and the patients would keep their masks on. No COVID-19 polymerase chain reaction test was required. We rescheduled patients telephonically; if they had COVID-19 symptoms, they were not allowed in the hospital. We limited the number of procedures per day (Fig. 2).

Especially during the third wave in South Africa, in June–September 2021, hospital beds in the hard-hit Gauteng province were unattainable. The province suspended elective surgery for another 7 weeks, adding to the extant waiting lists. This reinstitution of minor hand procedures assisted with a resolution to the prolonged waiting time for surgery.

### Prospects: private practice and outreach

Learning to incorporate WALANT in surgical practice is a gradual process. It requires patience but, in addition, adds to workflow efficiency. Carpal tunnel and trigger finger releases are procedures that are easy to perform in the early stages. The established cost-benefit at 1MH ensures the longevity of WALANT at SAMHS. However, private medical aid administrators are slowly “buying in” to WALANT procedures in consultation rooms from the so-called “in-hospital” benefits. This revolution is a new perspective in private health care in South Africa.

Essential life support resuscitation equipment and basic emergency medications are required as part of your practice’s initial set-up costs to ensure patient safety. We recommend having adequate light, a hand table, and an adjustable bed height. The vast possibilities of low costs and low risks make WALANT a sustainable method to limit health care expenditures in the long run, especially in low income countries.

## Conclusion

The possibilities with WALANT are increasing, with the procedure maintaining patients’ best interests and generating capacity to improve the surgeon’s horizons in the field. The satisfaction of a pain-free patient, who is able to contribute to the procedure and experience a finger that suddenly works, makes wide-awake surgery a game changer. In a country like South Africa, that buckles under economic pressure and has patients disheartened by the long waiting lists and the COVID-19 pandemic, wide-awake surgery offers hope for minor hand procedures. Flexibility, open mindedness, and resilience are necessary for the successful implementation of wide-awake surgery initiatives.
